# An Artificial Neural Network for Nasogastric Tube Position Decision Support

**DOI:** 10.1148/ryai.220165

**Published:** 2023-02-01

**Authors:** Ignat Drozdov, Rachael Dixon, Benjamin Szubert, Jessica Dunn, Darren Green, Nicola Hall, Arman Shirandami, Sofia Rosas, Ryan Grech, Srikanth Puttagunta, Mark Hall, David J. Lowe

**Affiliations:** From Bering Limited, 54 Portland Place, 2nd Floor, London W1B 1DY, England (I.D., R.D., B.S.); Emergency Department (J.D., D.G., N.H., A.S., S.R., D.J.L.) and Department of Radiology (R.G., S.P., M.H.), Queen Elizabeth University Hospital, Glasgow, Scotland; and Institute of Health and Wellbeing, University of Glasgow, Glasgow, Scotland (D.J.L.).

**Keywords:** Neural Networks, Feature Detection, Supervised Learning, Machine Learning

## Abstract

**Purpose:**

To develop and validate a deep learning model for detection of nasogastric tube (NGT) malposition on chest radiographs and assess model impact as a clinical decision support tool for junior physicians to help determine whether feeding can be safely performed in patients (feed/do not feed).

**Materials and Methods:**

A neural network ensemble was pretrained on 1 132 142 retrospectively collected (June 2007–August 2019) frontal chest radiographs and further fine-tuned on 7081 chest radiographs labeled by three radiologists. Clinical relevance was assessed on an independent set of 335 images. Five junior emergency medicine physicians assessed chest radiographs and made feed/do not feed decisions without and with artificial intelligence (AI)-generated NGT malposition probabilities placed above chest radiographs. Decisions from the radiologists served as ground truths. Model performance was evaluated using receiver operating characteristic analysis. Agreement between junior physician and radiologist decision was determined using the Cohen κ coefficient.

**Results:**

In the testing set, the ensemble achieved area under the receiver operating characteristic curve values of 0.82 (95% CI: 0.78, 0.86), 0.77 (95% CI: 0.71, 0.83), and 0.98 (95% CI: 0.96, 1.00) for satisfactory, malpositioned, and bronchial positions, respectively. In the clinical evaluation set, mean interreader agreement for feed/do not feed decisions among junior physicians was 0.65 ± 0.03 (SD) and 0.77 ± 0.13 without and with AI support, respectively. Mean agreement between junior physicians and radiologists was 0.53 ± 0.05 (unaided) and 0.65 ± 0.09 (AI-aided).

**Conclusion:**

A simple classifier for NGT malposition may help junior physicians determine the safety of feeding in patients with NGTs.

**Keywords:** Neural Networks, Feature Detection, Supervised Learning, Machine Learning

*Supplemental material is available for this article.*

Published under a CC BY 4.0 license.

## Graphical abstract

**Figure:**
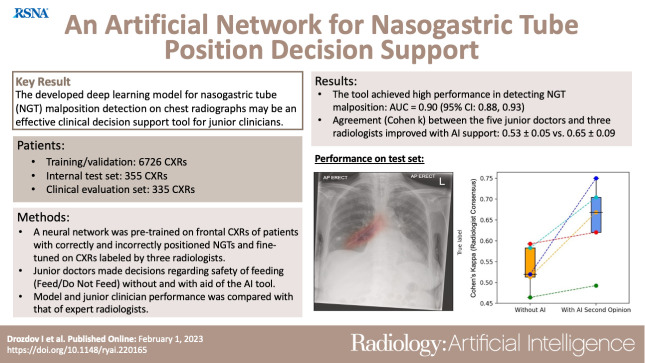

**Summary**

An artificial intelligence tool for detection of nasogastric tube malposition on frontal chest radiographs helped junior physicians make feeding decisions for patients.

**Key Points**

■ The nasogastric tube malposition detection tool achieved an area under the receiver operating characteristic curve of 0.90 (95% CI: 0.88, 0.93) compared with a consensus of three radiologists.
■ Mean agreement (Cohen κ) between five junior physicians and three radiologists regarding feeding decisions improved with artificial intelligence (AI) support (unaided vs AI-aided, 0.53 ± 0.05 vs 0.65 ± 0.09, respectively).

## Introduction

Nasogastric tube (NGT) feeding is an essential intervention that delivers enteral nutrition and medications when the oral route is insufficient or unsafe. The tube is inserted through the nostril, along the nasopharynx, through the esophagus, and into the stomach, ideally 10 cm below the gastroesophageal junction. More than 1 million and 800 000 NGTs per year are inserted in the United States and in the National Health Service population, respectively (1,2).

The rate of complications of bedside NGT insertions is 2%–36% (3,4), with inadvertent placement in the respiratory tract as the primary risk. pH testing of gastric aspirate is the first-line method to confirm NGT placement, but an aspirate may not always be immediately available in as many as 51.4% of patients because of factors such as adhesion of gastric mucosa to the tip of the NGT (5). Furthermore, gastric pH can be altered by commonly used medications, including proton-pump inhibitors and histamine-2 receptor blockers (6).

Chest radiography remains the reference standard method for confirmation of NGT placement (7). However, image interpretation may be challenging, especially for junior physicians, with misinterpretation of bronchial NGTs on chest radiographs linked to missed pneumothoraxes, bronchoaspiration, and death (1). Furthermore, because of increasing radiology backlog and a 33% rate of short staffing among the radiologist workforce (8), expert reporting is not always available in fast-paced environments, such as the emergency department; thus, imaging interpretation is often left to the requesting physician.

Deep artificial neural networks have been applied extensively in radiograph analysis (9–12). They present attractive clinical decision support tools, contributing to improved diagnostic accuracies among emergency medicine clinicians (13) and radiologists (12). Despite their general use, artificial neural network performance in dynamic real-world systems can be disrupted because of concept drift, caused by extrinsic factors such as changes in imaging workflows, instrument calibration, or software updates (14). Furthermore, application of artificial neural networks to detection of NGT malposition has been limited to detection of bronchial placements (15) and NGT segmentation (16). To our knowledge, a formal assessment of an artificial neural network model as a clinical decision support system to determine safety of feeding in patients with NGTs has not yet been undertaken.

This study had the following aims: *(a)* to develop a classification model for NGT malposition detection on chest radiographs, *(b)* to define a simple data-driven approach to assess model sensitivity to concept drift detection, and* (c)* to test the hypothesis that artificial intelligence (AI)-assisted NGT interpretation yields improved agreement (compared with unassisted assessment) between junior emergency department physicians and board-certified radiologists with decisions regarding safety of NGT feeding in patients (feed/do not feed).

## Materials and Methods

This retrospective study was funded, in part, by Bering Limited in the form of salaries to three authors (I.D., R.D., and B.S.). Authors who were not employees of Bering Limited had control of inclusion of any data and information that might present a conflict of interest for those authors who are employees of or consultants for that industry.

Delegated research ethics approval for this study (reference: 104690/WP6/S1) was granted by the Local Privacy and Advisory Committee at National Health Service Greater Glasgow and Clyde. Cohorts and de-identified linked data were prepared by the West of Scotland Safe Haven at National Health Service Greater Glasgow and Clyde. In Scotland, patient consent is not required when routinely collected patient data are used for research purposes through an approved Safe Haven (17). For that reason, informed consent was not required and was not sought. All research was performed in accordance with relevant guidelines and/or regulations.

### Data

**Figure 1:**
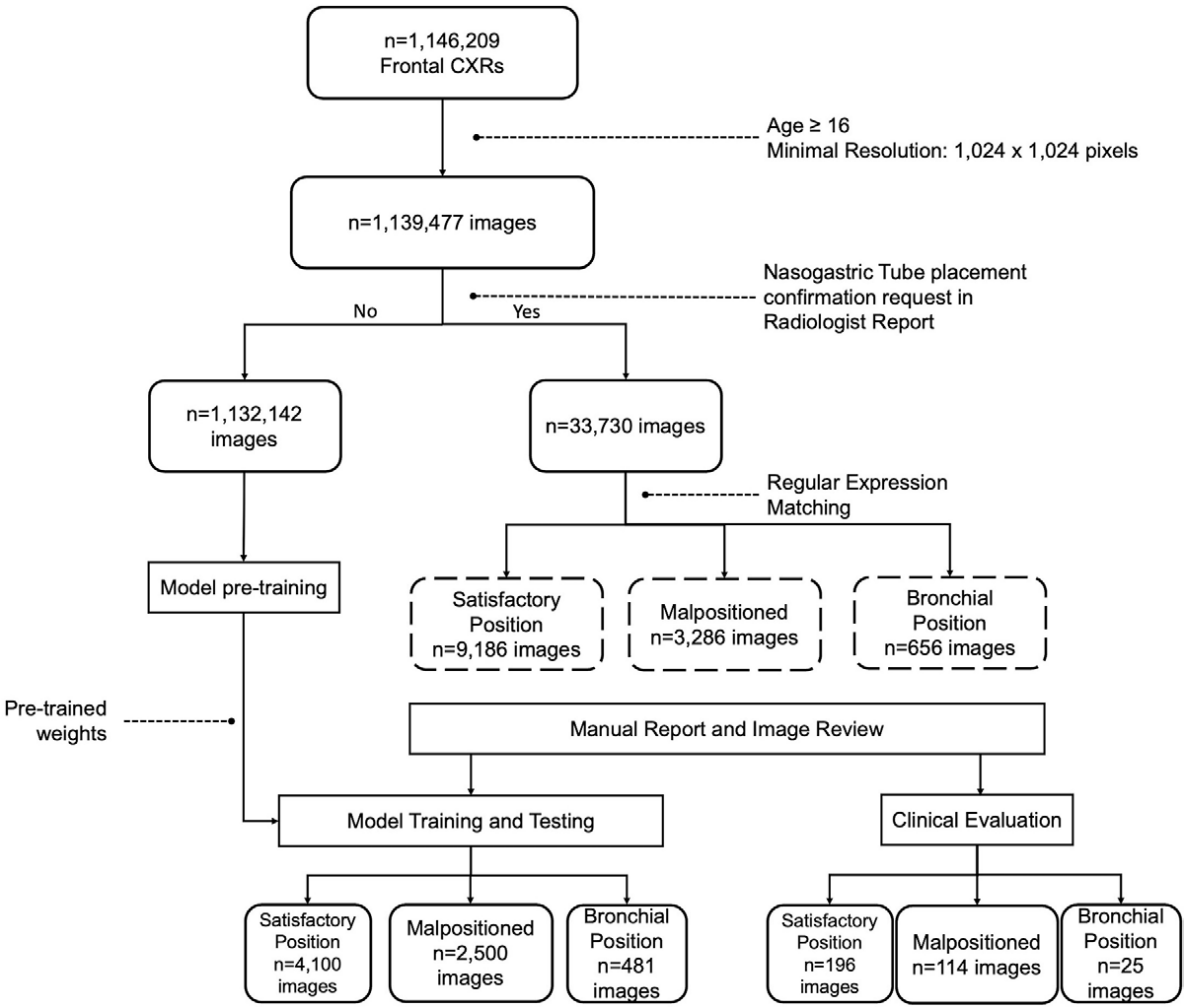
Flowchart shows the dataset selection process. Age and resolution filtering was applied to 1 146 209 frontal chest radiographs (CXRs). Of these, 1 132 142 images were selected for neural network pretraining, and 33 730 images were manually retained for training nasogastric tube positioning models. Nasogastric tube models were trained and tested on 7081 manually labeled chest radiographs (model training and testing set), and model efficacy as a feed/do not feed decision support tool was assessed on a separate set of 335 chest radiographs labeled by three radiologists and five junior physicians (clinical evaluation set).

All chest radiographs (Fig 1) (*n* = 1 146 209) were obtained between June 2007 and August 2019 across 14 acute sites in NHS Greater Glasgow and Clyde. Images were produced in a Digital Imaging and Communications in Medicine (DICOM) format by 11 different radiography systems, including those used for portable studies. Image resolution ranged from 253 × 902 to 4280 × 3520 pixels, with each pixel represented in gray scale with 16-bit precision. Identifiable patient data were removed from DICOM files and corresponding radiology reports using Named Entity Recognition algorithms within the Canon Safe Haven Artificial Intelligence (AI) Platform. This platform is a trusted research environment constructed specifically for machine learning within the health board network and deployed in National Health Service Greater Glasgow and Clyde through the Industrial Centre for AI Research in Digital Diagnostics, or iCAIRD.

Before analysis, DICOM images underwent stringent quality control procedures (Fig 1). First, images with width and height less than 1024 pixels were excluded from the study. Second, DICOM Body Part Examined (0018, 0015) and View Position (0018, 5101) attributes were filtered by “chest” and anteroposterior (“AP”) or posteroanterior (“PA”), respectively. Pediatric (patients < 16 years) chest radiographs were excluded from this study. Next, imaging studies explicitly requesting radiology review of NGT position confirmation (*n* = 33 730) were further filtered using regular expression matching any of the following keywords: “nasogastric,” “tube,” “placement,” “insertion,” and “bronchus”; 13 758 images were retained as candidates for manual annotation. All chest radiographs containing the keyword “bronchus” in the linked radiology report (*n* = 887) were manually reviewed to confirm a bronchial placement. Remaining chest radiographs were manually annotated in chronologic order based on their StudyDate DICOM tag (0008, 0020).

Overall, 4100 correctly placed NGTs, 2500 malpositioned NGTs, and 481 chest radiographs with bronchial NGT insertions were used in model training and testing. An additional set of 335 chest radiographs that contained correctly placed (*n* = 196), malpositioned (*n* = 114), and bronchial (*n* = 25) NGTs was identified from the remaining unlabeled images, composing the clinical evaluation set (Fig 1). Ground truth frequencies in the clinical evaluation set matched those in the training set. Remaining DICOM images were used in neural network pretraining.

### Ground Truths and Data Partitioning

All images were labeled by three board-certified radiologists (R.G., S.P., and M.H., who were 8 months to 5 years after fellowship of the Royal College of Radiologists Examinations; average experience of 3.2 years). A total of 7081 images used in model training and validation were labeled as “satisfactory position” (the tip of the tube is clearly visible in the stomach, at least 10 cm below the gastroesophageal junction, and is safe for feeding), “malpositioned” (not in the stomach and not in the lungs, trachea, or bronchus), or “bronchial insertion” (the tube has entered the trachea and right or left main bronchus). Three hundred thirty-five images used for clinical model evaluation were labeled by the three radiologists as “feed” (the tip of the tube is clearly visible in the stomach, at least 10 cm below the gastroesophageal junction) or “do not feed” (position is unclear or malpositioned, or the tube has entered the bronchial tree). Consensus radiologist labels were generated using majority voting. Images for neural network pretraining were labeled as “normal” or “abnormal” (defined as the presence of one or more radiologic signs; Table S1) by processing linked free-text radiology reports with a custom transformer neural network (Fig S1) (11,18).

Images were randomized into stratified training (90%), validation (5%), and testing (5%) sets with preserved frequencies of ground truth labels, sex, and view positions. To avoid data leakage, we ensured that patient identifiers did not overlap between splits.

### Model Training

Classification ensemble constituents used a modified InceptionV3 architecture (19). Input layers were adjusted to accept 764 × 764-pixel and 1024 × 1024-pixel resolution DICOM files. The classification head consisted of a global average pooling layer, followed by a dense layer with rectified linear unit (20) activation and a dropout layer. A softmax activation function was applied to the final dense layer. Model ensemble output probabilities were calculated by averaging probabilities of each constituent model.

The number of neurons in the penultimate dense layer and the dropout rate were tunable hyperparameters optimized during training using the Hyperband algorithm (21), with the best set of parameters corresponding to the lowest categorical cross-entropy loss on the validation set. The number of neurons was selected from the range of [32, 512], and the dropout rate took values from the range [0, 0.2].

Models were trained in two phases. In the first phase (pretraining), networks were initialized with ImageNet weights (22) and trained to differentiate normal and abnormal chest radiographs using transformer-generated ground truths from 1 132 142 DICOM images and linked radiology reports (Fig 1). In the second phase (classification), models were initialized with the pretrained weights and further fine-tuned on manually annotated NGT DICOM images.

Training was performed with 32 images per batch using an Adam optimizer with a learning rate of 1 × 10^−3^ while minimizing the categorical cross-entropy loss. Input images were resized to 764 × 764 or 1024 × 1024 pixels using bilinear interpolation without preserving the aspect ratio. During training, images were subject to random augmentations, which included brightness adjustments, angular rotation, and left-to-right flipping. Training was terminated early if validation loss did not improve after 10 consecutive epochs.

Details regarding the methods used for detection of captured features and concept drift detection are in Appendix S1.

### Clinical Study Design

Model use as a clinical decision-support tool was assessed on additional 335 images. This was a three-phase study. In phase 0 (ground truth), three radiologists, described above, reviewed all chest radiographs, making a feed/do not feed decision using their prior experience viewing NGT placement. In phase 1 (baseline), five junior emergency department physicians (each with 2–5 years of clinical experience; average, 3.5 years) assessed NGT positions and made a feed/do not feed decision based on chest radiograph view only. In phase 2, images were randomized, and AI-generated NGT position probabilities (satisfactory, malpositioned, and bronchial) were positioned above the chest radiograph. Junior physicians were asked again to make a feed/do not feed decision while considering model outputs and their own clinical judgment. There was a 1-month delay between phases 1 and 2. All images were reviewed using software (LabelStudio version 1.4.1, Heartex; *https://labelstud.io*).

### Statistical Analysis

Saliency maps were generated using gradient-weighted class activation mapping (23) of the final convolutional layer. Model performance was assessed using area under the receiver operating characteristic curve (AUC), overall accuracy, sensitivity, specificity, and positive predictive value. For AUC measures, 95% CIs were calculated empirically using 2000 bootstrap samples. CIs for sensitivity, specificity, and accuracy are exact Clopper-Pearson CIs. Interobserver agreement was measured using Cohen κ. Differences in mean κ values between radiologists and junior physicians with and without AI support were assessed using the two-tailed Student *t* test. Patient demographic characteristics, image characteristics, and data sources of the NGT positioning model were compared across the training and validation, internal testing, and clinical evaluation sets using analysis of variance for continuous variables and χ^2^ for categorical variables. *P* values less than .05 were considered to indicate a statistically significant difference. All statistical tests were performed using the SciPy module (version 1.7.3) for Python (version 3.9.14).

## Results

### Patient Characteristics

Patient age and sex distributions were similar between training, testing, and clinical evaluation sets, with a mean age range of 66.3–67.4 years ± 14.9–18.9 (SD) and a male-to-female ratio of 1.4–1.5:1 (Table 1).

**Table 1:**
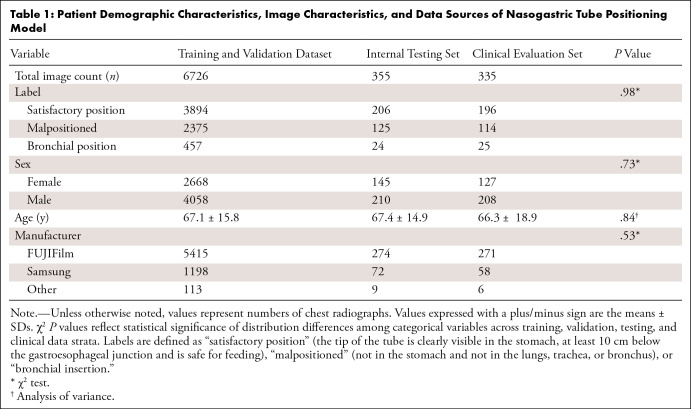
Patient Demographic Characteristics, Image Characteristics, and Data Sources of Nasogastric Tube Positioning Model

### Detection of NGT Malposition

**Figure 2:**
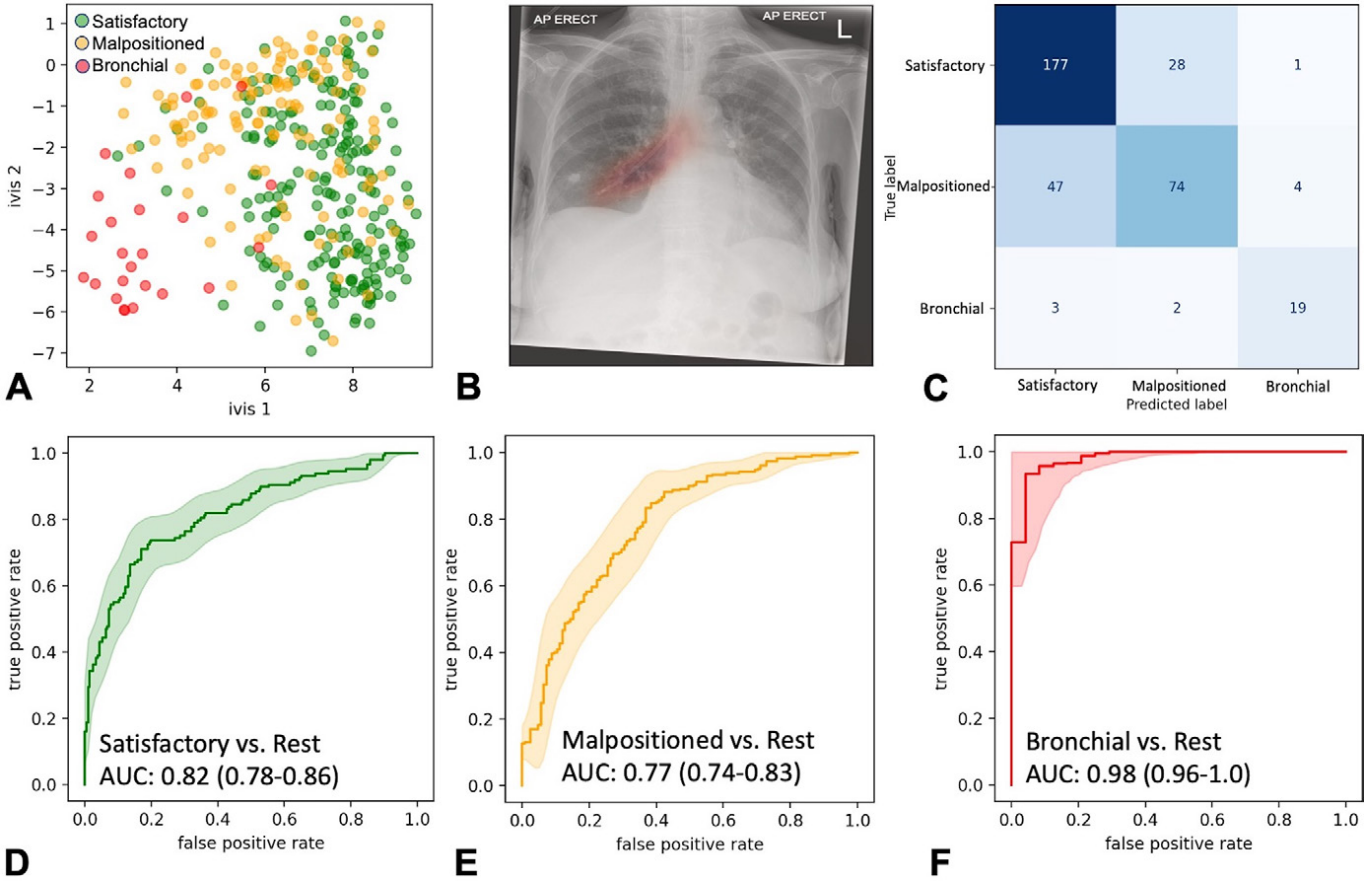
Ensemble performance for nasogastric tube (NGT) malposition detection on the testing set (355 images). **(A)** Scatterplot shows two-dimensional twin neural network (Ivis) embedding of the combined global average pooling layer values in the NGT malposition ensemble. Each point represents a single chest radiograph in the testing set. Green, orange, and red points reflect satisfactory, malpositioned, and bronchial NGT ground truth values, respectively. **(B)** Heatmap shows gradient-weighted class activation mapping activation of the final convolutional layer in the 1024 × 1024 InceptionV3 model superimposed over a bronchial-positioned NGT. **(C)** Ensemble confusion matrix between ground truths and predicted image labels. Predicted labels reflect the class with the greatest classification probability. **(D–F)** Receiver operating characteristic curves for each class of interest. Shaded areas are 95% CIs, generated using 2000 bootstrapped samples. AP = anteroposterior, AUC = area under the receiver operating characteristic curve.

The low-resolution (764 × 764 pixels) and high-resolution (1024 × 1024 pixels) models of our ensemble trained for 16 and 19 epochs, respectively, before reaching early stopping criteria. Dimensionality reduction of the global average pooling layers and chest radiograph saliency maps confirmed model propensity to learn the target class (Fig 2A, 2B). The model ensemble achieved AUCs of 0.82 (95% CI: 0.78, 0.86), 0.77 (95% CI: 0.71, 0.83), and 0.98 (95% CI: 0.96, 1.00) for satisfactory, malpositioned, and bronchial positioned NGTs, respectively, in the testing set (*n* = 355 images) (Fig 2C–2F). The ensemble classifier exhibited improved class probability calibrations compared with its constituents (Fig S2).

The best performance was observed in bronchial NGT classifications, achieving an accuracy of 97% (345 of 355; 95% CI: 95%, 99%), positive predictive value of 79% (19 of 24; 95% CI: 61%, 90%), sensitivity of 79% (19 of 24; 95% CI: 58%, 92%), and specificity of 98% (95% CI: 97%, 99%) (Table 2).

**Table 2:**
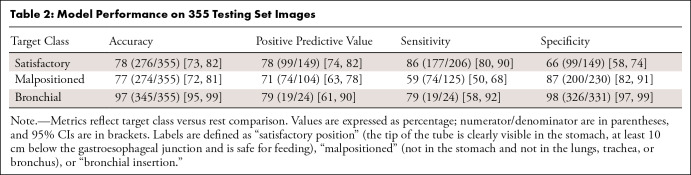
Model Performance on 355 Testing Set Images

**Figure 3:**
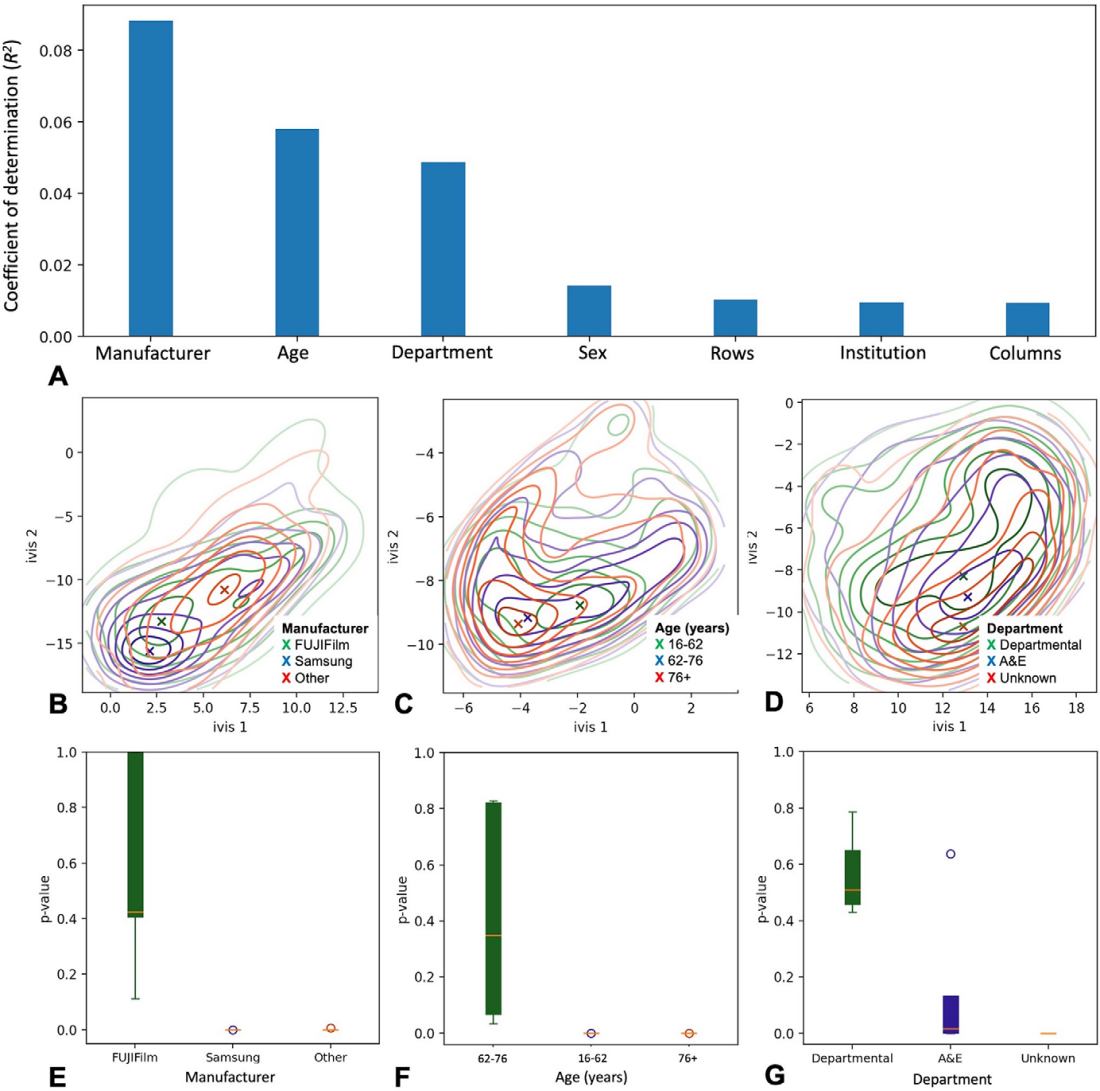
Captured model features and concept drift detection. **(A)** Bar chart shows Digital Imaging and Communications in Medicine (DICOM) tags and their respective *R*^2^ values. Values reflect variance within the global average pooling layer explained by each DICOM tag. **(B–D)** Contour plots represent two-dimensional embedding of global average pooling layer values in the testing set. Colors correspond to individual confounder values, and density centroids are shown as an X. **(E–G)** Box and whisker plots of two-sample Kolmogorov-Smirnov test *P* values show likelihood of concept drift in a testing set of interest. Green boxes are testing set samples with the same inclusion criteria as the training set, and blue and red boxes are testing set samples with known concept drift (red boxes very small due to tight *P* values). The box extends from the lower to upper quartile values of the data, with a line at the median. The whiskers extend from the box to show the range of the data, bounded by the fifth and 95th data percentile. Points represent values past the end of the whiskers. A&E = accidents & emergency.

Salient chest radiograph metadata features captured by our model were identified by calculating the coefficient of determination (*R*^2^) between low-dimensional representations of the global average pooling layer and numerically encoded DICOM tags of interest (see Materials and Methods). Manufacturer (0008,0070), patient age (expressed as the number of days between study date [0008,0020] and birth date of the patient [0010,0030]), and institutional department name (0008,1040) cumulatively explained 20% of variance in the global average pooling embeddings (Fig 3A), whereas class label itself accounted for 58%.

Misclassifications made by the model ensemble were interpretable. For example, all five images incorrectly classified as bronchial NGTs were from patients who underwent gastric pull-up surgery, with NGT observed within the supradiaphragmatic stomach (Fig S3A). Similarly, 22 of 28 (79%) satisfactory NGTs classified as malpositioned were noted within the hiatus hernia (Fig S3B). Distinction between malpositioned and correctly placed NGTs was the source of most confusion for the model, with 47 of 121 (38.8%) images incorrectly classified as satisfactory. Manual review of the 47 false-negative radiographs identified that in all cases, NGTs required advancement by only 1–3 cm.

### Concept Drift Awareness

We combined out-of-bag drift probabilities of a random forest classifier with a two-sample Kolmogorov-Smirnov test to monitor concept drift in a series of out-of-sample testing sets (see Appendix S1). In all cases, out-of-sample AUCs were adversely affected (Table S2), and statistically significant drift was detected when images with sudden changes in either radiograph system manufacturer, patient age, or institutional department name were introduced to the model (*P* < .001; Kolmogorov-Smirnov test) (Fig 3E–3G). The model was most sensitive to images obtained using different equipment manufacturers (*P* = .41 [no drift] vs *P* < .001 [drift]; Kolmogorov-Smirnov test). Similarly, for models trained on patients who were aged 62–76 years, a sudden shift to chest radiographs from younger patients (16–62 years) and older patients (≥76 years) resulted in statistically significant drift of class-probability estimates (*P* < .001; Kolmogorov-Smirnov test). The effect was also present, albeit to a lesser degree, in models trained on departmental images (*P* = .52; mean Kolmogorov-Smirnov test) with a sudden shift to inference on images acquired in the emergency department (*P* = .05; mean Kolmogorov-Smirnov test).

### Model as a Second Opinion for Junior Physicians

**Figure 4:**
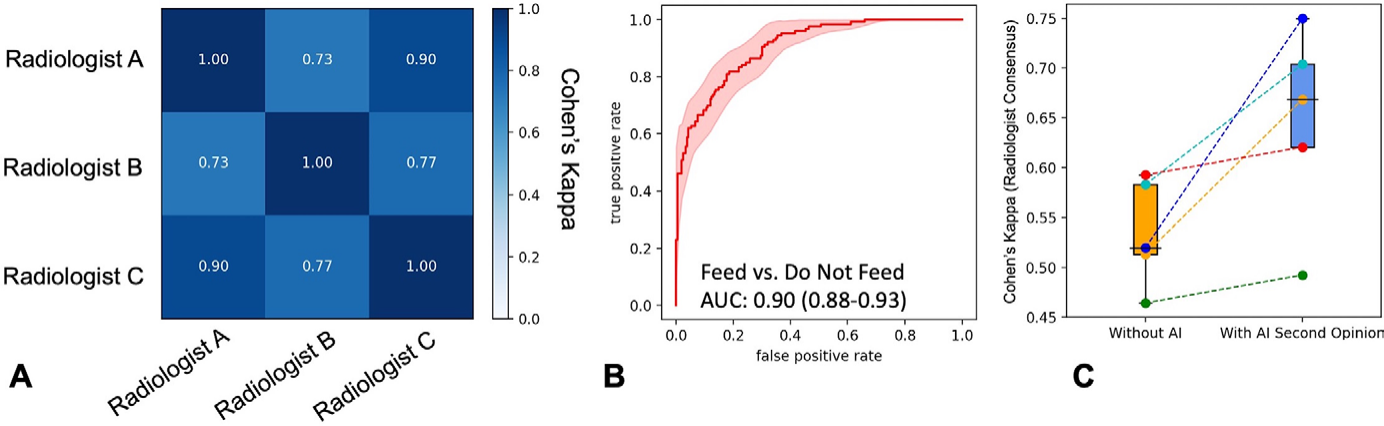
Effect of nasogastric tube malposition detection model on feed/do not feed decisions of junior physicians. **(A)** Heatmap shows feed/do not feed interradiologist decision agreement as Cohen κ values. **(B)** Receiver operating characteristic curve of model performance compared with consensus radiologist feeding decision. Shaded region is 95% CI. **(C)** Box plots show agreement between junior physicians and consensus radiologist feeding decision without (orange) and with (blue) artificial intelligence (AI) decision support. The box extends from the lower to upper quartile values of the data with a line at the median. The whiskers extend from the box to show the range of the data, bounded by the fifth and 95th data percentile. Points are individual observations and dotted lines are the magnitude of change in κ values for individual clinicians. AUC = area under the receiver operating characteristic curve.

In an independent set (335 images), mean interradiologist Cohen κ and percentage agreement for feed/do not feed decision were 0.87 ± 0.11 and 90% ± 3.4, respectively (Fig 4A, Table S4). The model ensemble, compared with consensus of the three radiologists, achieved an AUC of 0.90 (95% CI: 0.88%, 0.93%), with positive predictive value, sensitivity, and specificity values of 73% (95% CI: 65%, 81%), 73% (95% CI: 68%, 83%), and 83% (95% CI: 79%, 85%), respectively (Fig 4B).

Interreader agreement among junior physicians was 0.65 ± 0.03 at baseline and 0.77 ± 0.13 following AI-assisted decision support. The mean agreement between junior physicians and radiologists at baseline was 0.53 ± 0.05. With AI decision support, performance improved (two-sided *t*-statistic, 2.24; *P* = .05), reaching mean agreement with radiologist consensus of 0.65 ± 0.09 (Fig 4C). Similarly, overall accuracy, positive predictive value, sensitivity, and specificity improved by 3.7%–12.3%, with the greatest percentage change in positive predictive value (12.3%) (Fig S4, Table 3).

**Table 3:**
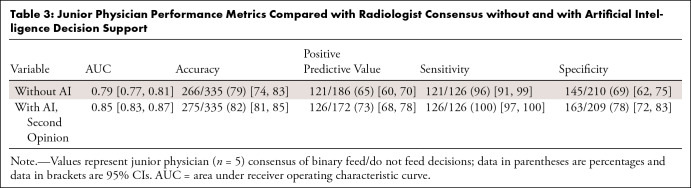
Junior Physician Performance Metrics Compared with Radiologist Consensus without and with Artificial Intelligence Decision Support

## Discussion

NGT feeding is an essential intervention. However, the procedure can be complicated by inadvertent tube placement in the respiratory tract (3,4). We demonstrate that a simple deep learning model for detection of NGT malposition on frontal chest radiographs increases the average agreement between junior emergency department physicians and radiologists from 0.53 ± 0.05 (unaided) to 0.65 ± 0.09 (AI-aided) (two-sided *t*-statistic, 2.24; *P* = .05).

The NGT malposition classifier is an ensemble of InceptionV3 artificial neural networks modified to accept 764 × 764-pixel and 1024 × 1024-pixel images. Previous studies (9,11) showed that averaging the predictions from two artificial neural networks operating at different spatial resolutions yielded the best performance. It is likely that features learned by multiresolution networks are complementary, with each network capturing features missed by the other. In addition, although neural network probabilities are known to be poorly calibrated (24), probabilities obtained from NGT ensemble were better calibrated compared with individual models. This is likely because of a reduction in model variance following probability averaging (25).

Previous models have detected bronchial NGTs with an AUC of 0.87 (compared with an AUC of 0.98 for our ensemble) (15), whereas endotracheal tube malposition was detected with an AUC of 0.99 (26). Considering the relatively small training set sizes (*n* = 5475–16 000), our higher model performance is likely due to artificial neural network weight initialization. It is well understood that initialization with ImageNet weights compared with random weights results in higher performance on domain-specific tasks (15,27). In this work, we pretrained all ensemble constituents using a large dataset of 1 132 142 frontal chest radiographs, labeled as normal or abnormal by a natural language processing algorithm. This approach has been demonstrated to improve transfer learning capacity and model robustness against imbalance (28). Interestingly, our classifier appeared to be specific for NGTs in the presence of endotracheal tubes, correctly labeling 57 chest radiographs with both tube types, of which two were bronchial NGTs.

We developed a data-driven strategy to delineate the salient features captured by our models by computing the coefficient of determination (*R*^2^) between low-dimensional representations of the model global average pooling layer and numerically encoded DICOM tags of interest. Although a number of algorithms exist to explain black box models (29,30), they are limited to tabular data. Our approach attempts to explain features captured within the unstructured imaging data using DICOM tags. As expected, the class label itself accounted for the most variance (58%) in global average pooling embeddings. Surprisingly, imaging features associated with manufacturer, patient age, and institutional department name accounted for over 20% of variance, suggesting that this information may be encoded at the pixel level. Artificial neural networks have been used to predict patient age using frontal chest radiographs (31,32), but differences in device calibration exist between manufacturers and hospital departments. It is unlikely that these features are confounders, given the low correlation between DICOM tags and NGT position labels (*R*^2^ = 0.07); however, our results suggest that exposure to diverse datasets, capturing a range of equipment, sites, and patient ages during training, is a critical requirement for model use.

Of particular interest was assessment of how model performance changes over the model’s life cycle. Although traditional performance drift approaches involve monitoring a metric (eg, AUC), this is not practical in a health care setting (14). We introduced a simple real-time drift detector that uses low-dimensional embeddings of the latent model space and calculates an interpretable drift *P* value using the two-sample Kolmogorov-Smirnov test at an image level. Our method enables real-time monitoring of model performance in the absence of contemporaneous ground truths. It is conceptually similar to the method used by Soin et al (14); however, it does not use a variational autoencoder to detect changes in input data, and instead uses a twin neural network (Ivis) to represent latent model information. As such, it can be easily adapted to nonimaging data. Through controlled experimentation, we have demonstrated that our model can be particularly sensitive to changes in chest radiography device manufacturer, patient age, and department where chest radiographs were acquired. The information provided by our drift statistics could inform model monitoring, auditing, retraining, and redeployment.

An important methodologic consideration in this article is the reliance on board-certified radiologists to provide the ground truth labels. Consequently, the trained model becomes a mechanism by which expertise can be conveyed to junior physicians. Use of our deep learning model as a decision support tool improved the feed/do not feed agreement between junior physicians and radiologists from 0.53 ± 0.05 (unaided) to 0.65 ± 0.09 (aided). Poststudy interviews revealed that junior physicians were more confident in making this decision with AI support. This was corroborated by an increase in an overall accuracy of a decision to feed from 69% (unaided) to 78% (aided). The decrease in false-positive findings (*n* = 65 [unaided], *n* = 46 [aided]) suggests that our model could reduce the number of complications associated with NGT reinsertions by as much as 29.2% (33). Of note, whereas five bronchial NGTs were incorrectly assessed by unaided junior physicians as safe for feeding, there were no incorrect feeding decisions with AI support. Overall, our results suggest that improvements in safety and performance can be achieved through synergistic decision support in fast-paced clinical environments (14).

Interreader agreement among junior physicians also improved from 0.65 ± 0.03 (unaided) to 0.77 ± 0.13 (aided with AI). Use of the artificial neural network as a decision support tool probably reduces ambiguity in chest radiograph misinterpretation and may alleviate potential anchoring bias (34).

Our study had several limitations. First, it was limited to classification of NGT malposition and did not include segmentation analysis to allow for tube localization. Second, the clinical evaluation was not carried out on a typical radiologist workstation. Although the environment was the same for all radiologists and junior physicians, clinicians were unable to manipulate images to the extent that radiology software would allow. Third, the retrospective nature of this study resulted in a level of class balance that may not represent real-world prevalence. Finally, because lateral chest radiographs are not routinely obtained in Scotland, there were no lateral images in this study. Further work will need to be carried out to assess model generalizability in other countries and the effect of lateral views on sensitivity and specificity of NGT malposition classification.

In conclusion, the developed deep learning tool for detection of NGT misplacement on chest radiographs may aid junior physicians in clinical decision-making regarding safety of feeding for patients with NGTs. Future work will include formal development and benchmarking of our drift detection system with tools such as CheXStray (14). In addition, we plan to introduce an NGT segmentation component to the ensemble. This would allow for visual localization of the course of an NGT on a given chest radiograph. This may lead to more rapid clinical image reviews, reducing interpretation time per image. Second, integration with a radiologist environment such as the picture archiving and communication system would enable clinicians to manipulate images as they do in routine practice and provide a more accurate account of the change in performance of junior physicians. Finally, a prospective study would allow for evaluation of the ensemble when exposed to real-world class distributions, assessing its effect on workflow safety and operational efficiency.
